# Supplementary material for: Machine Learning Approach to Predict Physical Properties of Polypropylene Composites: Application of MLR, DNN, and Random Forest to Industrial Data
Source: Polymers (Basel). 2022 Aug 26;14(17):3500. doi: 10.3390/polym14173500 (PMC9459971; doi:10.3390/polym14173500)
Supplement: Supplementary file 1 [file polymers-14-03500-s001.zip › polymers-1831991-supplementary.pdf]

SUPPLEMENTARY MATERIAL

Machine Learning Approach to Predict Physical Properties of Polypropylene Composites: Application of MLR, DNN, and Random Forest to Industrial Data

Chonghyo Joo [1,2,†], Hyundo Park [1,2,†], Hyukwon Kwon [1,2], Jongkoo Lim [3], Eunchul Shin [3], Hyungtae Cho [1] and Junghwan Kim [1,*]

[1] *Green Materials and Processes R&D Group, Korea Institute of Industrial Technology, 55, Jongga-ro, Jung-gu, 44413 Ulsan, Korea*

[2] *Department of Chemical and Biomolecular Engineering, Yonsei University, 50, Yonsei-ro, Seodaemun-gu, 03722 Seoul, Korea*

[3] *Research & Development Center, GS Caltex Corporation, 359, Expo-ro, Yuseon-gu, 34122 Daejeon, Korea*

[†] **Co-first authors: These authors contributed equally to this work.**

[*]Corresponding author

Junghwan Kim

E-mail: kjh31@kitech.re.kr

Table S1. Pseudo code of categorization

Algorithm: Categorization

Input: $\mathcal{M}$: $\mathrm{n} \times d$ recipe dataset, n: Number of recipes, d: Number of materials.

Output: C: Category set.

Binning process

for each $u \in \mathcal{M}$ **do**

  if $u > 0$ // If u is bigger than zero, replace the value of u with "T."

    replace u value with "T"

  if $u = 0$ // If u is zero, replace the value of u with "F."

    replace u value with "F"

end

$\mathcal{M} \rightarrow \mathcal{M}'$ // Recipe dataset replaced with "T" and "F" after binning process.

Categorization process

for each $u \in \mathcal{M}'$ **do**

  arrange u from u (n, 1) to u (n, d) // Make code a consisting of "T" and "F."

  give the arranged code a to data point (n,) // Give code a to each recipe.

for each code $a \in \mathcal{M}'$ **do**

  for each code $b \in \mathcal{M}' \setminus u$ **do**

    if $a = b$ // If a and b are same, add the recipes to the same category

      add (a, b) pair to the same category in the category set C

Return C
